# Nanoparticle-ultrasound synergy: an emerging theranostic paradigm for breast and gynecologic cancers

**DOI:** 10.3389/fonc.2025.1617939

**Published:** 2025-12-04

**Authors:** Tian Yang, Yelin Lou, Zhuopeng Ying

**Affiliations:** 1 Department of Ultrasound, JinHua Maternal and Child Health Care Hospital, Jinhua, China; 2 Department of Ultrasound, Affiliated Jinhua Hospital, Zhejiang University School of Medicine, Jinhua, China

**Keywords:** ultrasound imaging, ultrasound therapy, nanoparticles, theranostics, gynecologic oncology

## Abstract

Breast cancer (BC), cervical cancer (CC), and ovarian cancer (OC) are among the most prevalent and life-threatening malignancies affecting women worldwide. While conventional therapies have improved patient outcomes, they often result in suboptimal survival and quality of life. In recent years, ultrasound (US) has emerged as a promising therapeutic tool, not only for its well-established role in diagnostic imaging but also for its safety, deep tissue penetration, and real-time capabilities. The integration of US with nanotechnology has further expanded its potential, enabling nanoparticles (NPs) to function as contrast agents, drug delivery vehicles, and energy mediators in cancer theranostics. This review explores the synergistic effects of NPs and US in the diagnosis and treatment of breast and gynecologic cancers, with a focus on OC and CC, while also including BC due to its clinical significance and shared imaging modalities. We examine the biophysical mechanisms underlying US-based therapies, the design of multifunctional nanoplatforms, and their applications in enhanced imaging, high-intensity focused ultrasound (HIFU), sonodynamic therapy (SDT), and US-triggered drug delivery. Finally, we discuss the translational challenges and future prospects of these innovative technologies, emphasizing their potential to transform the clinical management of BC, CC, and OC.

## 1 Introduction

Cancer remains a leading threat to global health, necessitating the development of novel therapeutic strategies to overcome the limitations of conventional treatments (1). Nanotechnology has emerged as a transformative tool in biomedicine, enabling breakthroughs in diagnostics and targeted drug delivery (2).

The development of multifunctional nanoplatforms that integrate imaging and therapeutic capabilities represents a significant advancement in theranostic medicine (3). Ultrasound (US) technology offers distinct advantages for cancer management, including its excellent safety profile, real-time imaging capability, and clinical practicality. These features make it particularly valuable for managing both breast and gynecologic cancers (4–6). Recent advances have substantially expanded US applications through nanoparticle (NP)-enhanced approaches, enabling more precise interventions such as targeted drug delivery, sonodynamic therapy (SDT), and high-intensity focused ultrasound (HIFU) for tissue ablation. The strategic combination of US with nanotechnology creates powerful synergies that address multiple limitations of conventional cancer therapies. This integrated approach enhances both diagnostic accuracy and treatment efficacy while demonstrating strong potential for clinical translation (3).

**Figure 1:**
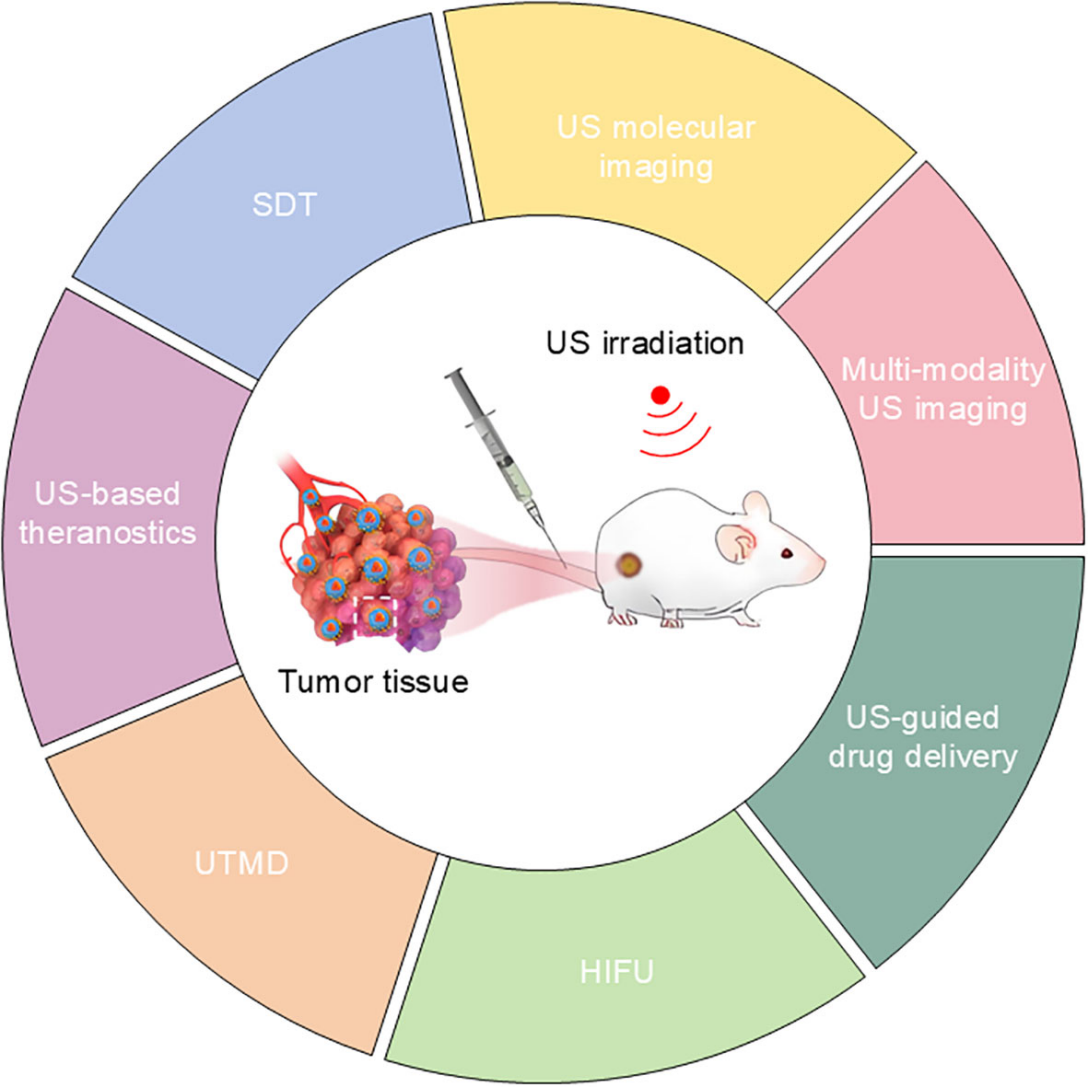
Nanoplatform-based ultrasound application structural framework.

This review provides a comprehensive analysis of NP-enhanced US applications in breast and gynecologic malignancies, including CC and OC. We examine current treatment modalities, fundamental biophysical mechanisms of US, and recent advances in NP-enhanced technology. The integration of cutting-edge nanotechnology with established US techniques shows considerable promise for developing more effective diagnostics and personalized treatments for these cancers (**Figure 1**).

## 2 Current approaches and diagnostic limitations

### 2.1 Breast cancer

BC is the most prevalent malignancy affecting women worldwide (7). It primarily arises from uncontrolled ductal cell proliferation that can develop into benign or metastatic tumors following carcinogen exposure (8). Current treatment protocols rely heavily on hormone therapies, particularly estrogen blockers such as tamoxifen and aromatase inhibitors (letrozole, anastrozole, and exemestane) (9). However, triple-negative breast cancer (TNBC), an especially aggressive subtype, presents significant therapeutic challenges. Current approaches are limited primarily to chemotherapy and anti-VEGF monoclonal antibodies like bevacizumab (10, 11). The emergence of nanomedicine has revolutionized BC treatment strategies, offering improved imaging capabilities and targeted therapeutic approaches. Cutting-edge molecular biology techniques facilitate the design of sophisticated multifunctional nanostructures capable of delivering chemotherapeutic agents, particularly in drug resistant cases. A recent review indicates promising nanotheranostic strategies for TNBC. These include a CRISPR-Cas detection system and engineered CAR-T cells with nanoliposomal viral vectors to target hypoxic CSCs. Engineered liposomes and polymeric NPs show particular promise for personalized treatment approaches through selective targeting of BC cells to induce apoptosis, offering new potential for managing refractory TNBC cases (12).

### 2.2 Cervical cancer

CC is the fourth most common gynecologic malignancy globally, accounting for over 300, 000 deaths annually (13). Treatment modalities include radiotherapy, surgical resection, and chemotherapy. Platinum-based agents like cisplatin and paclitaxel are commonly used (14–16). However, these conventional approaches face significant challenges, including high recurrence rates (35%) and substantial treatment-related toxicities such as multidrug resistance (MDR) and systemic organ damage (17, 18). In this context, nanobiotechnology has emerged as a promising therapeutic alternative. Various nanostructures—including metal-based, carbon-based, lipid-based, and polymer-based NPs—demonstrate considerable potential for improving CC treatment outcomes (19). These novel approaches aim to overcome the limitations of traditional therapies while minimizing adverse effects.

### 2.3 Ovarian cancer

OC remains one of the most aggressive gynecologic malignancies, with mortality rates exceeded only by CC (13). While current treatment modalities, including surgical debulking, platinum-based chemotherapy, and radiotherapy continue to evolve (20), their clinical efficacy remains limited by several critical challenges (21). Surgical interventions carry significant risks of peritoneal and abdominal lymph node metastasis, often leading to severe postoperative complications and increased mortality (22). Chemotherapeutic approaches, though widely employed, are hampered by substantial toxicity profiles and the development of drug resistance. Radiotherapy demonstrates restricted applicability and often fails to achieve durable remission, with repeated treatments potentially compromising progression free survival (23, 24). These persistent challenges highlight the critical need for innovative therapeutic strategies. NP-enhanced US approaches, especially those integrating imaging with targeted therapeutic delivery, provide a transformative framework for real-time treatment monitoring and precision oncology in OC management (21, 25).

## 3 Biophysical effects of ultrasound

**Figure 2:**
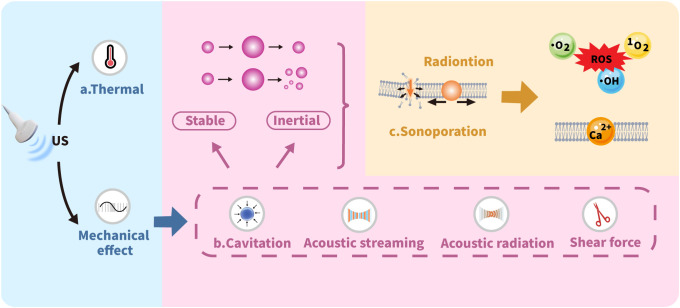
Mechanisms of ultrasound biophysical effects.

US imaging serves as a widely adopted clinical tool for disease diagnosis and assessment. This technology offers significant advantages including noninvasiveness, cost-effectiveness, and real-time imaging capability, while providing deep tissue penetration without ionizing radiation exposure (26–29). The therapeutic potential of US is mediated through distinct thermal and mechanical mechanisms, with key processes illustrated in **Figure 2**.

### 3.1 Thermal effect

The thermal effects of US primarily arise from the temperature increase in the medium due to US energy absorption. The generated heat is directly proportional to both the wave frequency and exposure time while inversely proportional to the specific absorption coefficient of the target tissue. Consequently, media with higher absorption coefficients exhibit more pronounced temperature elevations, leading to more significant thermal effects on tissues (30, 31). Elevated temperatures typically induce changes in the fluidity of cell membrane phospholipid bilayers, generally enhancing membrane permeability to nanomedicines. Even moderate temperature increases can induce structural deformation in NPs. This may progress to complete disruption through US-based expansion, ultimately triggering NPs rupture that facilitates localized drug release. Such thermally responsive materials, commonly composed of polymers or lipids, called as heat-sensitive NPs, which US can activate to improve targeted drug release. Additionally, US-triggered thermal effects enable localized thermal ablation, particularly through HIFU application (32).

### 3.2 Cavitation

Cavitation significantly enhances sonoporation effects when microbubbles (MBs) are introduced into the cellular microenvironment. MBs serve as contrast agents to improve US image quality. To achieve optimal imaging and therapeutic outcomes while minimizing risks, careful adjustment of US frequency and intensity is required to preserve bubble integrity. Beyond imaging, MBs enable targeted drug delivery by using US to selectively disrupt bubbles at specific sites, triggering controlled drug release. Cavitation occurs in two distinct forms: stable cavitation and inertial cavitation. Stable cavitation features sustained, nonlinear oscillations of gas-filled MBs around an equilibrium radius. This process continues until the encapsulated gas fully dissolves into the bloodstream and is cleared through pulmonary exhalation. Inertial cavitation presents a markedly different behavior, defined by the catastrophic implosion of MBs that happens immediately when US is applied (32–35).

### 3.3 Sonoporation

Sonoporation refers to the US induced formation of transient pores in cell membranes through mechanical interactions. This process physically compromises membrane integrity, creating micropores that facilitate passive entry of drug molecules, NPs, and genetic material into cells (36). Cavitation plays a pivotal role in sonoporation by generating gas-filled MBs that undergo creation, oscillation, and eventual disintegration under US exposure. When US waves interact with MBs, they induce high-frequency oscillations that produce fluid shock waves and exert shear forces on cell membranes. These mechanical effects alter membrane structure and significantly enhance permeability, thereby improving cellular uptake of therapeutic NPs (32).

## 4 Clinical application of US

### 4.1 Nanoplatforms for US imaging

The growing demand for early disease detection and diagnosis continues to drive innovations in imaging technologies and contrast agent development. The ongoing advancement of NPs as imaging contrast agents holds significant promise for diverse clinical applications in the future (36). Their appeal in biomedical imaging stems from multifunctional targeting capabilities-encompassing passive, active, and physical targeting mechanisms. A key advantage lies in their nanoscale dimensions, which facilitate the enhanced permeability and retention (EPR) effect in tumor tissues. This property significantly prolongs contrast agent retention at target sites and markedly improves imaging efficacy (37). While conventional MB contrast agents (typically 1-10 μm in diameter) remain the clinical gold standard for blood pool imaging in contrast-enhanced ultrasound (CEUS), their inability to extravasate beyond the vasculature limits their utility for extravascular targeting. Recent years have consequently witnessed the rise of nanoscale ultrasound contrast agents (UCAs) as a transformative approach for molecular US imaging (6). Consequently, nanoscale UCAs have emerged as a transformative platform for molecular US imaging. These nanoplatforms enable precise visualization of tumor tissues at the molecular level, and their representative designs and applications are systematically summarized in **Table 1**.

**Table 1:** Nanoplatforms for ultrasound imaging.

Cancer type	Nanoparticle	Size	Effect	Ref
Breast Cancer	NBs	478.2 ± 29.7 nm	• This ND showed good US enhancement, displaying a peak intensity of 104.5 ± 2.1 dB under US contrast scanning.	(40)
	NBs-Her	613.0 ± 25.4 nm	• NBs-Her improves the quality of contrast-enhanced US imaging and also penetrates tumor tissue for extravascular imaging, but does not penetrate normal skeletal muscle.	(41)
	MSNs	1-30 µm	• Increasing US image contrast.	(43)
	Silica NP	200 nm	• It can be used as a highly effective contrast agent for color Doppler US imaging of human breast tissue.	(44)
	Cur-NDs-2	101.2 nm	• Even at low concentrations, chitosan nanodroplets produce a strong ultrasonic contrast through the droplet-to-bubble transition.	(45)
	DiR-MB	0.4-6 μm	• US-targeted DiR-MB disruption and then conversion to DiR-NP identifies and directs inaccessible cancer lesions.	(46)
	Na2CO3/Fe3O4@PLGA/ Cy5.5/RGD NPs	117.6 nm	• This pH-responsive NP system provides good effects in MR/US/fluorescent imaging.	(51)
Cervical Cancer	AB-NB	74.6 ± 16.7 nm	• OVCAR-3 tumors showed higher peak US signal intensity and lower washout compared to CA-125-negative SKOV-3 tumors. Targeted MBs also exhibited increased tumor retention and prolonged echogenicity compared to untargeted NBs.	(42)
Ovarian Cancer	FA-OINPs	300 nm	• It has good contrast as a US/PA contrast agent both *in vivo* and *in vitro*.	(54)

#### 4.1.1 US molecular imaging

The engineering of molecularly specific antibodies or ligands onto ultrasound contrast agents (UCAs) creates targeted nanoplatforms—including nanobubbles, echogenic liposomes, and nanodroplets—that enable molecular ultrasound imaging by specifically binding to disease markers through ligand-receptor interactions. This advancement transforms ultrasound from a conventional anatomical imaging tool into a molecular-targeted modality, where functionalized UCAs serve as the fundamental component by concentrating in diseased tissues and enhancing lesion-specific signals. Nanoscale UCAs further excel in tumor imaging due to their superior vascular penetration via the EPR effect and modifiable surfaces for improved target retention, with each variant offering distinct imaging advantages (6, 38, 39). The comparison between various UCAs used in molecular US imaging is summarized in **Table 2**.

**Table 2:** Comparison of ultrasound contrast agents for molecular ultrasound imaging.

Type	Size	Core	Shell	Advantage	Disadvantage
Nanobubble	<1μm	Perfluorocarbon Gas	Lipid, Polymer	• Small size • Potential for long circulation	• Weaker Signal than microbubbles • Poor physical stability • Difficult fabrication
Microbubble	1-10 μm	Perfluorocarbon Gas	Lipid, Albumin, Polymer	• Strong Signal, clinical gold standard • Well-established technology	• Large Size • Short circulation half-life
Gas Vesicle	45-800nm	Air	Protein	• Ultra-stable	• Potential immunogenicity
Nanodroplet	<1μm	Perfluorocarbon Liquid	Lipid, Polymer, Surfactant	• Activatable via phase-change • Capable of tissue extravasation for enhanced targeting	• Requires high-energy ultrasound for activation • Risk of embolism from spontaneous phase change • Weak echo signal in liquid state
Echogenic Liposomes	100–500 nm	Gas or Perfluorocarbon Liquid/Gas	Phospholipid Bilayer	• Mature liposome platform • Easy functionalization	• Weaker and less stable acoustic signal • Challenges in consistent gas/liquid loading

Recent advances in NP-enhanced UCAs have substantially improved imaging capabilities. Gas-filled nanobubbles (NBs) show particular promise with their surfactant-stabilized phospholipid shells and fluorocarbon cores. Yang et al. pioneered HER2-targeted NBs using biotin-streptavidin interactions, achieving enhanced US signals despite immunogenicity limitations (40). Jiang et al. subsequently improved this approach through covalent herceptin conjugation, creating more stable HER2-targeted NBs that maintained tumor specific signal enhancement for 40 minutes (41). Further developments have expanded the UCA repertoire. Gao et al. developed CA125 antibody-coupled NBs demonstrating twofold greater signals in OC xenografts (42). Milgroom’s team created herceptin-functionalized mesoporous silica NPs for BC imaging (43), while Martinez et al. designed gas-filled hollow silica NPs that enhance Doppler imaging (44).

Innovative approaches continue to emerge. Baghbani et al. introduced phase transitioning curcumin-loaded chitosan nanodroplets (45), and Lin et al. developed a convertible DiR-MB system for intraoperative tumor visualization (46). These technologies collectively advance US from conventional imaging toward precise cancer diagnosis and image-guided therapy. Enhanced stability, improved specificity, and multifunctionality characterize this new generation of contrast agents.

#### 4.1.2 Multimodal US imaging

Multimodal US imaging represents a major diagnostic advance by integrating complementary techniques to provide comprehensive molecular level information (47). This strategy leverages the unique advantages of individual imaging methods while overcoming their limitations through nanotechnology-enabled probes (48). In cancer diagnosis and treatment monitoring, combining photoacoustic (PA), US, and magnetic resonance imaging (MRI) within multifunctional nanoplatforms shows particular promise (49). These systems utilize PA’s excellent optical contrast, US’s real-time capability, and MRI’s superior soft tissue resolution, creating synergistic platforms that outperform single modality approaches and enable more accurate tumor evaluation (49).

Integrating MRI and US combines their complementary strengths: MRI provides high spatial resolution for deep tissues, while US enables real-time imaging. Together they improve sensitivity and reduce acquisition time (50). Nanocontrast agents further enhance this approach by boosting each modality’s sensitivity and enabling targeted tumor visualization (50). For example, Nie et al. developed a pH-responsive NP system that generates CO_2_ in acidic tumor environments. This produces strong US echoes while enabling excellent MRI and fluorescence imaging (51). This smart platform demonstrates the potential of combining MRI/US/fluorescence through tumor microenvironment responsive agents (51).

PA imaging generates acoustic waves through laser induced photothermal effects, detected and converted into PA signals. Combined with US, it synergistically enhances imaging by leveraging PA’s high optical selectivity with US’s deep penetration and resolution (52, 53). Liu et al. created folate receptor-targeted NPs (FA-OINPs) that demonstrated robust US/PA contrast enhancement in both cellular systems and OC xenografts (54). This platform successfully enabled image guided therapy, showing strong potential for clinical translation (54).

### 4.2 Nanoplatform-based US targeted therapy

Recent advances in NP-enhanced US have expanded its applications in oncology, including drug delivery, HIFU, and SDT (55–57). Engineered NPs leverage their small size and surface properties to achieve tumor targeting via the EPR effect and specific tumor microenvironment interactions (58). These NPs enhance US imaging, improve SDT efficacy, augment HIFU ablation, and enable controlled drug release (56, 59, 60). Representative therapeutic nanoplatforms are summarized in **Table 3**. Furthermore, they can integrate multiple imaging and therapeutic functions into single theranostic platforms (61). These combined systems, detailed in **Table 4**, enable simultaneous diagnosis and treatment while providing real-time monitoring capabilities. The integration of different modalities in these nanoplatforms significantly improves treatment precision and therapeutic outcomes.

**Table 3:** Nanoplatforms for ultrasound-based therapy.

Cancer type	Nanoparticle	Size	Effect	Ref
	Nanodroplets	39.2 ± 3 nm	• In the BC mice models, US-mediated therapy with doxorubicin-loaded PFH nanodroplets showed excellent anti-cancer effects characterized by tumor regression.	(63)
	PFP/ICG/DOX@LIP	362.23 nm	• Such PFP nanodroplets with phase/size tunable properties enable site-specific drug delivery efficiently and exhibit their potent in cancer theranostics.	(64)
Breast Cancer	CLs	81 ± 2 nm	• The efective utilization of liposomes as a delivery system for curcumin, thereby ofering a promising avenue for targeted breast and other cancer treatments.	(67)
	TRA-liposomes	101.10 ± 1.13 nm	• Exposing the liposomes to LFUS triggered drug release which increased with the increase in power density.	(68)
	FA-PL-dMSN	169 ± 47 nm	• Localized enhancement of the mechanical effects of HIFU in cancer cells.	(71)
	Lip-ABC	174.8 ± 58.26 nm	• In an *in vitro* synergistic HIFU ablation of mammary tumors in bovine liver and BALB/c nude mice, Lip-ABC outperformed controls.	(74)
	Catalase@MONs	145.9 nm	• It can serve as both a contrast agent for US-guided HIFU tumor ablation and a HIFU synergist.	(75)
	Mn-MOF	70 nm	• It catalyzes the production of O_2_ from H_2_O_2_ to alleviate tumor hypoxia, while decreasing the expression of GSH and GPX4, thereby enhancing SDT and producing better antitumor effects in H22- and 4T1-loaded mice.	(82)
	T80(T-ce6/PL)	18.28 ± 8.49 nm	• Intracellular ROS production was significantly elevated in MCF-7 human BC cells after US exposure.	(85)
	BFIP	114 nm	• BFIP demonstrates superior carrier separation over bismuth fluoride and generates abundant ROS under US. It also depletes glutathione via oxidative pathways, disrupting the TME through oxidative stress.	(86)
	LDH-MTX@CMM-Ce6	225.1 ± 9.5 nm	• This formulation achieves homologous BC targeting and induces potent apoptosis by combining MTX-mediated cell cycle arrest with ultrasound-triggered SDT.	(87)
	IR780@LD-Fe3O4/OA	217.35 ± 13.53 nm	• Providing accurate and effective SDT for MDR BC.	(88)
	CpMBs	2.02 ± 1.5 μm	• Enhancement of siRNA transfection efficiency and porphyrin uptake in MCF-7 cells by sonoporation effect.	(94)
	siHIF@CpMB	1-7 μm	• Ultrasound-triggered siHIF@CpMBs-to-NPs conversion boosts tumor delivery of porphyrin/siRNA through cavitation.	(95)
	ABCG2-siRNA-loaded PEAL NPs	131.5 ± 6.5 nm	• ABCG2-siRNA-loaded NP with UTMD effectively silenced the ABCG2 gene and enhanced ADR susceptibility in MCF-7/ADR (ADR-resistant human cancer cells).	(96)
	(G5-TPGS@y-CDs)-DOX	279.2 nm	• It can overcome the MDR of cancer cells and effectively inhibit the growth of cancer cells and tumors.	(92)
	SLNs	450.9 nm	• The TNO variant it produces can be used as a potential stimulus response platform for site-specific delivery of chemotherapeutic agents such as 5-FU for the treatment of CC.	(66)
Cervical Cancer	PEI-FA-DSTNs	173 ± 15 nm	• It is a potent drug delivery system that, in combination with SDT, enhances the anticancer activity of curcumin.	(83)
	AIBA@MSN	80–100 nm	• Under hypoxic conditions, azo radicals induce tumor cell death via the non-oxygen radical pathway.	(84)
	Bubble liposomes	500nm-1μm	• Bubble liposomes in combination with US are an excellent non-viral vector system for IL-12 cancer gene therapy.	(65)
	Fe3O4@SiO2-Ce6, FSC	460 nm	• It improves the efficacy of SDT and applies it to the *in vitro* treatment of OC.	(80)
Ovarian Cancer	SIM@TR-NPs	119.1 ± 1.9 nm	• The platform converts cold to hot tumors via ICD and TAM modulation, showing potent ovarian cancer suppression and TME reprogramming.	(89)
	PSP@MB	500 nm	• US biophysical effects with PSP@MB delivery of ALDH1-shRNA promotes OCSC apoptosis.	(93)

**Table 4:** Nanoplatforms for combined imaging and therapy.

Cancer type	Nanoparticle	Size	Effect	Ref
Breast Cancer	PCSTD-Gd	309.5 ± 39.4 nm	• CSTD enhances tumor targeting via amplified EPR effects, boosts MRI sensitivity with a higher r1 relaxation rate, improves gene delivery through efficient DNA compaction and serum resistance, and increases drug loading capacity.	(103)
	Doc-PFH@SL@PD-HA	248.07 ± 5.74 nm	• Enhancing US imaging signals and facilitating drug release.	(98)
	LP@PFH@HMME	105 ± 1.64 nm	• This nanoplatform enables robust 19F MRI/CEUS bimodal imaging, offering excellent aqueous solubility, a long T2 relaxation time (1.072 s), and high 19F content for sensitive 19F MRI. It also promotes effective nanomedicine accumulation after intravenous injection.	(99)
	AS1411-PLGA@FePc@PFP	201.87 ± 1.60 nm	• This dual-mode PA/US contrast agent enables precise diagnostic imaging and therapy guidance, while exhibiting effective NIR-triggered heating for potent antitumor effects *in vitro* and *in vivo*.	(100)
	LIP3	200 nm	• It features active targeting, bimodal imaging, visualization of drug release, and precise treatment in the presence of LIFU.	(101)
	Her2-GPH NPs	282.3 nm	• Her2-functionalized NPs significantly enhanced US/MRI molecular imaging of target cells. It acts as an effective light absorber and specifically induces SKBR3 cell death under near-infrared laser irradiation.	(102)
Cervical Cancer	PFeRu-PL@SiO2(R) NPs	225 nm	• It enables MRI/US dual-modality imaging-guided synergistic HIFU for CC.	(105)
Ovarian Cancer	AIPH-MSTN@BSA-MnO2@CCM	225.9 ± 1.7 nm	• US-triggered AIPH decomposition amplifies TiO2-mediated ROS/imaging, while CCM coating enables targeted tumor delivery through homologous targeting and immune evasion.	(104)

#### 4.2.1 US- guided drug delivery

Beyond imaging, US enables targeted drug delivery by triggering controlled release from NPs and enhancing tumor penetration (32). Its deep tissue reach and safety make US ideal for activating NPs carrying drugs, genes or proteins (3, 62). US enhances therapy through three primary mechanisms: releasing drugs from NPs, increasing tumor NPs uptake, and improving tumor penetration (32).

State-of-the-art US-triggered nanocarriers show remarkable potential for targeted oncological interventions. Baghbani et al. developed adriamycin-loaded alginate nanodroplets that showed both enhanced anticancer effects and durable US contrast under sonication (63). Sheng et al. confirmed the potential of tunable nanodroplets for US guided BC treatment (64). Suzuki’s team achieved successful IL-12 gene delivery to tumors using bubble liposomes combined with US, inducing local immune responses (65). Innovative approaches include thermo-sensitive nanoorganogels (TNOs) incorporating solid lipid NPs (SLNs) for 5-FU release in CC under thermal/US stimulation (66), and curcumin-loaded liposomes that exhibited improved stability and antitumor effects when combined with US and MBs (67). Furthermore, Elamir et al. developed trastuzumab-functionalized immunoliposomes showing power dependent drug release under low frequency US (68). These US-based nanoplatforms enable focused drug release within the US target area, allowing for dose reduction and selective tumor targeting while minimizing systemic side effects (32, 65–68). Collectively, these advances highlight the transformative potential of US-triggered NP systems for precision cancer therapy.

#### 4.2.2 High-intensity focused ultrasound

HIFU is an emerging noninvasive ablation technique for localized tumors. By concentrating low energy US on target tissues, HIFU induces tumor cell necrosis through instantaneous thermal effects, cavitation, and mechanical forces (69). NP integration has significantly enhanced HIFU therapy. NPs improve ablation efficiency while minimizing damage to surrounding healthy tissues (3). Notably, modern multifunctional NPs serve dual roles as HIFU potentiating agents and diagnostic contrast agents, enabling precise image-guided ablation procedures (70).

NP-enhanced HIFU therapy improves tumor ablation precision and efficacy. Adem Yildirim et al. developed phospholipid capped mesoporous silica NPs (MSNs) that locally amplify HIFU’s mechanical effects in BC cells (71). Phase change NPs have further enhanced HIFU efficacy by modifying the acoustic microenvironment to improve energy deposition in tumors (72, 73). Feng et al.’s ammonium bicarbonate-loaded liposomes (Lip-ABC) demonstrated superior stability and tumor accumulation, significantly enhancing HIFU ablation in both ex vivo and *in vivo* models (74). Liu et al. created a catalytic nanoreactor (catalase@MONs) that serves as both UCA and HIFU synergist through oxygen generation (75). These nanoplatforms address key challenges in HIFU therapy by improving targeting precision, reducing required energy doses, and integrating diagnostic capabilities.

#### 4.2.3 Sonodynamic therapy

SDT combines low intensity US with specialized sensitizers to generate reactive oxygen species. This approach induces cancer cell death through oxidative damage and is particularly effective for deep seated tumors (76, 77). Traditional SDT faces limitations due to suboptimal sensitizer performance. Recent advances in nanomedicine have led to engineered nanosensitizers that enhance tumor accumulation, improve ROS generation efficiency, and enable multifunctional theranostic capabilities (78–80). NP-based systems overcome depth limitations by focusing US energy in deep tissues. They transform SDT into a clinically viable treatment option through enhanced tumor targeting and therapeutic performance (76–79, 81).

Significant advancements include Zhou et al.’s magnetically guided nanorobots that deliver sonosensitizers to OC sites (80). Xu et al.’s Mn-MOF nanosensitizers alleviate tumor hypoxia and suppress antioxidant defenses (82). Malekmohammadi et al. developed folate targeted silica titanium dioxide mesoporous NPs for combined chemo-SDT in CC (83) and Wang et al. designed AIBA@MSNs to induce immunogenic cell death in CC through non-oxygen-dependent pathways (84). Further innovations include Kang’s Tween 80-based nanocarriers for BC specific SDT (85), and Zhu’s bismuth-based heterostructures that synergize radiotherapy with SDT (86). Li’s cell membrane camouflaged platform enables chemo-SDT (87), while Shi’s lipid droplet-based system overcomes multidrug resistance in BC (88). Wang’s NPs combine SDT with immunotherapy to reprogram macrophages (89).These developments collectively demonstrate how engineered NPs are overcoming traditional SDT limitations by improving sensitizer delivery, enhancing ROS generation, enabling combination therapies, and integrating diagnostic capabilities for more effective cancer treatment.

#### 4.2.4 Ultrasound-targeted microbubble destruction

UTMD has emerged as a safe strategy for enhanced drug and gene delivery (62). This technology leverages dual mechanisms: increasing vascular and cellular membrane permeability through sonoporation, and facilitating *in situ* MBs-to-NPs conversion to improve cellular uptake and therapeutic outcomes (70). MBs serve as multifunctional US-based carriers. They enable targeted drug release and CEUS, enabling real-time treatment monitoring and precise spatiotemporal control of delivery (90). Their structure supports versatile cargo loading, accommodating drugs, genes, NPs, or therapeutic gases (91).

UTMD technology enhances cancer therapy through improved drug/gene delivery and real-time monitoring. Li et al. developed yellow fluorescent carbon dot/dendrimer nanocarriers that enhance drug uptake and efficacy in MDR BC via sonoporation effects (92). Similarly, PSP@MB NPs exhibit US-triggered, GSH-responsive properties for improved OC stem cell treatment through enhanced endocytosis (93). For gene therapy, Zhao’s porphyrin-grafted MBs deliver FOXA1-siRNA and convert to NPs under low frequency US (94). Sun et al. extended this approach using HIF1α-siRNA-loaded MBs (95). Bai’s team demonstrated UTMD-enhanced delivery of ABCG2-siRNA NPs can overcome chemotherapy resistance *in vivo* (96). These studies establish UTMD as a powerful platform for enhanced delivery, real-time imaging guidance, and overcoming treatment resistance.

#### 4.2.5 US-based theranostics

Nanotheranostic platforms combine imaging and therapeutic capabilities within single NP systems. These integrated approaches enable real-time monitoring of drug distribution while facilitating therapeutic intervention (97).

Mou et al. developed Doc-PFH@SL@PD-HA NPs that simultaneously enhance US imaging under NIR irradiation while delivering combined photothermal-chemotherapy (98). Chen’s LP@PFH@HMME liposomes enable bimodal imaging-guided SDT and immunotherapy in TNBC (99). He’s A-FP NPs achieve targeted BC therapy with integrated PA/US imaging (100). Zhao’s LIP3 nanoplatform exemplifies sophisticated design with active targeting, bimodal imaging, and visualized drug release under LIFU guidance (101). Further innovations include Dong’s HER-2-targeted nanocarriers for US/MRI and photothermal therapy (102), Gong’s Gd-based dendritic polymers for MRI-guided chemotherapy (103), and Xie’s dual-responsive AMBC platform overcoming hypoxia for US/MRI-guided SDT (104). Mai’s hybrid nanovesicles combine US/MRI imaging with HIFU-triggered chemotherapy to prevent tumor recurrence (105). These multifunctional nanosystems collectively represent a paradigm shift in cancer management. They enable real-time treatment monitoring, precision image-guided therapy, and synergistic combination of multiple treatment modalities.

## 5 Discussion

The integration of nanotechnology with US has established a powerful theranostic platform that synergistically combines the inherent advantages of US, including its safe, deep tissue penetration capability, and real-time imaging features, with the multifunctional properties of NPs. This convergence enables highly targeted therapeutic interventions coupled with real-time treatment monitoring, fundamentally reshaping management strategies for breast and gynecologic cancers. Accumulating preclinical evidence demonstrates the superior efficacy of these US-triggered multimodal approaches over conventional monotherapies, primarily achieved through enhanced and synergistic tumor suppression mechanisms. Particularly noteworthy is the unique value proposition of NP-enhanced US technology, which integrates real-time operation, cost-effectiveness, absence of radiation exposure, and exceptional deep tissue penetration capacity. These combined characteristics render this technology especially suitable for treating deep seated gynecological tumors such as OC located within the pelvic cavity, presenting a compelling alternative to conventional diagnostic and therapeutic modalities.

US imaging has been extensively validated in diverse preclinical models, driving the development of numerous targeted contrast agents that have significantly advanced our understanding of disease mechanisms. However, the transition to clinical application requires improved standardization of these agents, particularly in manufacturing and characterization processes. While targeted MBs like BR55 remain the current clinical gold standard due to their established safety profile, most nanoscale contrast agents still await large scale clinical trials to demonstrate human applicability. The translation pathway faces additional challenges in funding allocation, as industrial support typically focuses on late stage clinical trials, creating significant financial barriers for academic research investigating novel molecular targets. This funding gap substantially hinders the bridging of preclinical discoveries to clinical applications and slows the overall progress of US molecular imaging translation.

Beyond these validation and standardization hurdles, several critical barriers impede the clinical translation of NP-enhanced US theranostics. A primary concern lies in the comprehensive biosafety and long term toxicity profile of engineered NPs. While many systems show favorable biocompatibility in acute settings, their long term fate, including potential immunogenicity, off-target accumulation, and the clearance pathways of nondegradable components, demands thorough investigation. Concurrently, the regulatory pathway for such complex theranostic agents remains arduous. Regulatory bodies like the FDA have well established protocols for drugs or devices separately, but combination products present unique hurdles. Defining critical quality attributes, ensuring manufacturing reproducibility at scale, and establishing standardized characterization methods for these multifunctional systems are significant obstacles that must be overcome.

Further technical and translational barriers persist. The targeting efficiency of NPs in heterogeneous human tumors needs enhancement beyond the often unreliable passive EPR effect. For therapies like SDT, translating efficacy from small animal models to human patients requires overcoming challenges in US energy delivery at greater tissue depths while sparing healthy structures. Moreover, the field currently lacks standardized protocols for US parameters across different NP systems, hindering comparative analysis and clinical optimization. The inherent complexity of these platforms also raises the question of cost-effectiveness and scalability, which will be crucial for widespread clinical adoption.

Looking forward, overcoming these hurdles necessitates a concerted, interdisciplinary effort. Future research must prioritize the rational design of next generation “smart” nanoplatforms that respond specifically to the tumor microenvironment for precision action. There is also a compelling need to explore novel therapeutic mechanisms, such as the induction of unconventional cell death pathways like ferroptosis, which may help overcome treatment resistance. Expanding the repertoire of combination therapies to include partnerships with immunotherapy or gene therapy represents another fertile ground for innovation. Ultimately, the goal is to leverage the rich diagnostic data provided by these systems to create truly personalized treatment protocols, tailoring both US parameters and NP dosing to individual patient needs.

In summary, while the path to clinical translation is paved with challenges in safety, regulatory compliance, and standardization, the potential of NP-enhanced US therapeutics to revolutionize cancer care is undeniable. Through ongoing collaboration across materials science, biology, clinical oncology, and regulatory science, the field can advance these sophisticated nanoplatforms from promising prototypes to practical clinical tools, ultimately paving the way for more effective, personalized, and less invasive treatments for breast and gynecologic cancers.
